# A Brief Online Mentalization-Based Video-Feedback Intervention (VFI-RF) for Mother–Infant Interaction in Postnatal Risk Conditions: Protocol for a Multicenter Single-Arm Feasibility Study

**DOI:** 10.3390/jcm15135271

**Published:** 2026-07-06

**Authors:** Cristina Mazza, Francesca Favieri, Lucia Lombardi, Carmen Trumello, Eleonora Fiorenza, Michela La Stella, Anna Maria Della Vedova, Alessandra Babore, Renata Tambelli

**Affiliations:** 1 Department of Human Neurosciences, “Sapienza” University of Rome, 00185 Rome, Italy; cristina.mazza@uniroma1.it; 2 Department of Psychology, G. d’ Annunzio University of Chieti Pescara, 66100 Chieti, Italy; francesca.favieri@unich.it (F.F.); c.trumello@unich.it (C.T.); a.babore@unich.it (A.B.); 3 Department of Dynamic and Clinical Psychology and Health Studies, “Sapienza” University of Rome, 00185 Rome, Italy; eleonorafiorenza94@gmail.com (E.F.); renata.tambelli@uniroma1.it (R.T.); 4 Control-Mastery Theory Italian Group, 00179 Rome, Italy; michela.lastella@libero.it; 5 KOS Group, Villa Sant’Alessandro 00137 Rome, Italy; 6 KOS Group, Villa Armonia, 00165 Rome, Italy; 7 Department of Clinical and Experimental Sciences, University of Brescia, 25123 Brescia, Italy; anna.dellavedova@unibs.it

**Keywords:** telehealth, video-feedback, psychodynamic intervention, mentalization, parental reflective functioning, postnatal, preterm birth, perinatal anxiety, perinatal depression, maternal vulnerabilities

## Abstract

The postnatal period involves significant emotional and relational shifts that can challenge early mother–infant interactions, particularly under conditions of psychosocial vulnerability (e.g., maternal anxiety/depression) or infant-related risk (e.g., preterm birth). Maternal mentalization, operationalized as Parental Reflective Functioning (PRF), is a key protective factor for sensitive caregiving and dyadic regulation. Objectives: This protocol describes a multicenter, open-label, single-arm feasibility study evaluating a brief, fully online, mentalization-based video-feedback intervention (VFI-RF). The study is designed to assess the feasibility and acceptability of the intervention, rather than its efficacy. We aim to recruit 48 mothers, 24 in each of two risk groups, through socio-health services and neonatal intensive care units. Risk Group 1 will include mothers with clinically significant depressive and/or anxiety symptoms, defined as EPDS > 9 and/or GAD-7 ≥ 10, whereas Risk Group 2 will include mothers of preterm infants, defined as infants born before 37 weeks of gestation. Methods: The intervention consists of 8 + 2 synchronous online sessions over approximately 5 months. Mothers record brief everyday caregiving interactions (~5 min) to review with a trained clinician, focusing on the infant’s internal states and reflective meaning-making. Assessments occur at baseline (T0, infant age ~3 months), post-intervention (T1, ~8 months), and follow-up (T2, ~12 months). Primary feasibility outcomes include recruitment/referral metrics, uptake, retention, assessment completion, missing data, and participant-reported acceptability. Secondary exploratory clinical outcomes include maternal PRF, symptoms, parenting stress, social support, and mother–infant attachment, evaluated via validated self-report questionnaires. Results: The study is designed to evaluate referral and recruitment patterns, intervention uptake, and participant retention, as well as the acceptability and suitability of study procedures and outcome measures for a future controlled trial. Preliminary trajectories of change in maternal reflective functioning and early relational indicators will be examined descriptively and exploratorily. Conclusions: Findings will inform the feasibility and refinement of a brief online mentalization-based video-feedback intervention to support at-risk mother–infant dyads during the first postnatal year. Trial registration: Registered on Open Science Framework, osf.io/6g9ja, date of registration 4th March 2026.

## 1. Introduction

Motherhood experience is accompanied by profound somatic, emotional, and relational changes [1] that contribute to the high degree of psychological complexity of the perinatal period. The transformations associated with this phase are fundamental for shaping the early mother–infant dyadic relationship, particularly with respect to caregiving processes and emerging patterns of mutual regulation. These changes involve individual psychological adjustments in the mother and become highly relevant at the relational level, influencing how she perceives, organizes, and responds to the infant’s signals. In this sense, mentalization, defined as the ability to interpret behavior in terms of underlying feelings, intentions, and thoughts, plays a central role in supporting dyadic exchanges and the stabilization of a secure and growth-promoting relational environment [2]. In parenting, this ability has been operationalized through the construct of Parental Reflective Functioning (PRF), defined as the caregiver’s capacity to identify, reflect upon, and make meaning of mental states within mother–infant interactions [3]. Higher PRF was reported as associated with sensitive caregiving, more coherent interpretations of the infant’s behavior, and favorable socio-emotional outcomes for children [4].

A large body of evidence indicates that maternal vulnerabilities during the perinatal period, such as depressive symptoms, young maternal age, and preterm birth can compromise reflective functioning and the quality of early mother–infant exchanges [5,6]. These difficulties may reduce the mother’s ability to regulate her own emotions and to perceive the infant’s behavior as motivated by specific mental states, increasing the risk of disengaged or intrusive interaction patterns [3]. Such disruptions in early relational processes may contribute to later socio-emotional and attachment difficulties in children, negatively affecting developmental trajectories [7,8].

The present study focuses on two postnatal risk profiles that, although characterized by partially different mechanisms, may converge on relational vulnerability. In mothers with clinically significant anxiety and/or depressive symptoms, risk is primarily related to maternal affective distress. Depressive symptoms may reduce emotional availability, vitality, and sensitivity to infant cues, whereas anxiety may increase threat monitoring, uncertainty, and difficulties in tolerating ambiguous infant signals [7,8]. These processes may interfere with the mother’s capacity to interpret the infant’s behavior in terms of intentional and affective mental states [9,10]. By contrast, in mothers of preterm infants, risk is not necessarily driven by maternal psychopathology alone but may arise from infant-related and contextual factors, including medical vulnerability, early separation, hospitalization, parental fear, and the challenge of reading the cues of an immature or medically fragile infant [11,12].

Despite these differences, both profiles may affect a shared relational pathway: the mother’s capacity to remain emotionally available, observe the infant’s signals, tolerate uncertainty, and construct reflective meanings about the infant’s internal states. Thus, the inclusion of both groups is grounded in the assumption that postnatal relational vulnerability may emerge through different routes, while converging on partially overlapping difficulties in dyadic regulation, cue interpretation, and reflective meaning-making.

In light of the potential impact of postnatal risk conditions on maternal reflective functioning and early mother–infant relationships, preventive and early interventions have increasingly focused on supporting parents’ capacity to recognize, interpret, and respond to their infant’s cues in mental-state terms. Across this field, different intervention approaches have shown promise in enhancing reflective functioning and improving the quality of parent–infant interactions [13,14]. Standard approaches, such as family-centered care, kangaroo mother care, and multisensory developmental interventions, have been associated with improvements in bonding, maternal involvement, and parents’ sense of agency by targeting different components of the dyadic interaction [11,12]. These approaches are clinically valuable, particularly because they can be embedded within neonatal care. However, they often require in-person delivery and substantial institutional resources, and they may not specifically target maternal reflective functioning.

A promising strategy in this field may be video feedback (VF), a technique widely used across several integrated intervention models to support parenting. VF typically involves video-recording brief sequences of mother–infant interaction and reviewing them together with a trained professional, who supports parents in observing the interaction, identifying infant cues, and exploring the dyadic exchange through guided reflection. Systematic evidence has documented consistent improvements in key dimensions of caregiving and interaction quality—including maternal sensitivity, emotional regulation, and dyadic synchrony—as well as beneficial effects on child outcomes, such as attachment security, cognitive and socio-emotional development, and behavioral functioning [15,16,17,18,19]. From a mentalization-based perspective, VF is thought to operate by supporting mothers in recognizing the infant as a psychological agent and fostering their curiosity about the infant’s internal states. This process may in turn promote greater maternal sensitivity, affective attunement, and mentalization capacities [20,21]. It is important to distinguish between the evidence base for VFI and the more specific evidence base for approaching in fully remote, synchronous protocols. Previous evidence concerns VF delivered in face-to-face, home-visiting, clinical, or hybrid formats, or within broader multicomponent parenting interventions [22,23,24]. These studies provide an important rationale for using video-feedback to support parental sensitivity, reflective observation, and dyadic interaction, but they do not directly establish the feasibility or effectiveness of a stand-alone, fully online, synchronous, mentalization-based VFI in postnatal risk contexts. The COVID-19 pandemic further accelerated the transition from in-person to remote delivery of early parenting interventions. In this respect, emerging evidence indicates that telehealth-based VF approaches are both feasible and acceptable for families and may produce outcomes comparable to those achieved through traditional face-to-face formats [25,26].

More recent evidence [27] reinforces the value of stand-alone video-feedback interventions (VFIs) as an effective and flexible approach to supporting early mother–infant relational processes. VFIs are typically brief, cost-effective, and flexible, making them suitable for both preventive and clinical contexts [24,28]. Despite this evidence, several methodological gaps remain. The promising effects of VFIs on parenting and child outcomes are accompanied by inconsistent findings across several domains, including parenting stress and maternal representations [27]. Moreover, although it can be delivered remotely, increasing accessibility and reducing logistical barriers for mothers who are unable to access in-person services, the evidence base for fully remote, synchronous, mentalization-based video-feedback interventions in the postnatal period remains limited [24,27]. Specific challenges of these new approaches need to be examined, including difficulties in ensuring privacy during sessions, reduced clinician control over the therapeutic setting, and the need to establish a sufficiently secure and reflective therapeutic frame through an online medium [29]. Important implementation questions remain unresolved, including whether mothers are willing and able to record and share interaction videos from home, how privacy and confidentiality can be protected, and whether the selected assessment and intervention procedures are acceptable and sustainable for mothers in different postnatal risk conditions.

On this basis, a mentalization-based VFI may be particularly suited to address both shared and profile-specific relational vulnerabilities. The use of a common VFI-RF protocol does not imply that the mechanisms of risk are identical across groups; rather, the intervention targets a shared relational process—parental reflective functioning in everyday mother–infant interactions—while allowing the therapeutic work to be tailored to each dyad’s specific vulnerabilities, strengths, and interactional patterns.

Within this framework, a preliminary single-arm feasibility study represents an appropriate first step to evaluate whether the proposed VFI-RF can be meaningfully and safely implemented in postnatal risk contexts. Before testing its efficacy in a controlled trial, it is necessary to determine whether mothers are willing to engage with a fully online video-feedback format and whether reflective work can be sustained within a remote therapeutic setting. Feasibility evidence is therefore essential to refine the intervention procedures, address potential privacy and implementation barriers, and optimize the design of a future randomized controlled trial [30].

### 1.1. Research Question

This feasibility study aims to determine whether a brief, stand-alone, synchronous online mentalization-based video-feedback intervention (VFI-RF) can be successfully delivered and evaluated among mothers experiencing postnatal risk conditions.

Specifically, the study will address the following research questions:

1. Can eligible mothers be successfully identified, recruited, and retained across the study period?
2. Are the assessment procedures, including questionnaire completion and follow-up assessments, feasible and acceptable to participants?
3. Are the privacy, video-sharing, and data management procedures acceptable and implementable in a fully online format?
4. Do participant-reported satisfaction and qualitative feedback support the acceptability of the VFI-RF intervention and study procedures?

### 1.2. Primary Outcomes

The primary objective of this study will be to generate preliminary evidence on the feasibility and acceptability of delivering and evaluating the VFI-RF intervention for mothers at risk during the postnatal period. In line with the aims of a feasibility study, the primary outcomes focus on feasibility and acceptability indicators rather than intervention effectiveness.

Feasibility will be assessed through enrolment indicators, including the number of mothers referred, the proportion deemed eligible, and the proportion of eligible mothers who provide informed consent. Additional feasibility indicators include intervention uptake, measured as the proportion of VFI-RF sessions attended, retention at baseline and follow-up, reasons for ineligibility or withdrawal, assessment completion, and the extent of missing data on key measures.

Feasibility indicators will be calculated using predefined denominators. Specifically, the number of mothers referred will represent all mothers referred to the study by participating services or who self-refer following study advertisements. Eligibility rate will be calculated as the proportion of referred mothers meeting inclusion criteria. Consent rate will be calculated as the proportion of eligible mothers who provide informed consent. Intervention uptake will be calculated as the proportion of scheduled VFI-RF sessions attended. Retention rates will be calculated as the proportion of consented participants completing each assessment time point (T0, T1, and T2). Assessment completion rates will be calculated as the proportion of participants who complete the planned questionnaires at each assessment wave. All feasibility indicators will be reported for the overall sample and separately for each risk group (i.e., Risk Group 1: anxiety/depression; Risk Group 2: preterm birth).

Acceptability will be assessed using both quantitative indicators and qualitative feedback. The acceptability of the assessment procedures will be evaluated through participants’ views on the number, frequency, duration, content, and mode of delivery of study assessments. Mothers’ experience of the VFI-RF intervention and study procedures will be explored through in-depth feedback on their experiences, satisfaction, and perceived acceptability. Together, these quantitative and qualitative outcomes will be interpreted using pre-specified progression criteria based on a traffic-light system, to determine whether the intervention and study procedures would be suitable for a future larger-scale evaluation.

### 1.3. Secondary Outcomes

Secondary exploratory objectives include describing preliminary trajectories in maternal reflective functioning, maternal distress, perceived social support, maternal emotional bonding with the infant, and health-related quality of life in young children. These outcomes will be examined descriptively and exploratorily, with the aim of assessing the suitability and sensitivity of the selected measures for use in a future controlled trial, rather than testing intervention effectiveness.

By examining feasibility, acceptability, and preliminary clinical trajectories across different maternal risk conditions, this study will provide information on the practicality of delivering an exclusively online VFI-RF intervention during the postnatal period and on the suitability of the selected procedures and outcome measures for future randomized controlled trials (RCTs). In parallel, the study will explore potential barriers to implementation and to a subsequent larger-scale RCT, as well as determine what amendments and/or additional information may be required to optimize the design, delivery, and evaluation of the intervention in future trials. The practicality of implementing these changes will also be considered.

Overall, these findings will be used to determine whether a definitive main study is: (a) not feasible; (b) feasible pending modifications to the study protocol; or (c) feasible as originally designed.

### 1.4. Trial Design

This multicenter study is an open-label, single-arm feasibility study using a pre–post design with a nested qualitative evaluation. The VFI-RF will be delivered synchronously online by a trained clinician. Assessments will be conducted at three time points: baseline (T0; infant age ~3 months), post-intervention (T1; infant age ~8 months), and follow-up (T2; infant age ~12 months). Qualitative data will be collected through semi-structured interviews to further explore mothers’ experiences with the intervention and inform refinements for future research. The study protocol was drafted in accordance with the recommendations of the SPIRIT (Standard Protocol Items: Recommendations for Interventional Trials) statement. The corresponding checklist is available as Supplementary Materials.

## 2. Materials and Methods

### 2.1. Participants and Intervention

#### 2.1.1. Eligibility

The study will include mothers in the perinatal period who meet criteria for one of two at-risk groups: (1) mothers presenting clinically significant anxiety and/or depressive symptoms and (2) mothers who have given birth to a preterm infant, defined as birth before 37 weeks of gestation.

#### 2.1.2. Inclusion Criteria

The inclusion criteria for the general eligibility (applies to both groups) will be: (i) mothers in the perinatal/postnatal period aged ≥ 18 years; (ii) adequate knowledge of Italian; (iii) access to a digital device and a stable internet connection sufficient for online sessions and study assessments.

For Risk Group 1 (anxiety/depression), the inclusion criterion will be the presence of clinically significant anxiety and/or depressive symptoms, as identified through screening with the Edinburgh Postnatal Depression Scale (EPDS; cut-off score > 9) and the Generalized Anxiety Disorder 7-item scale (GAD-7; cut-off score ≥ 10). Accordingly, anxiety symptoms alone will qualify mothers for inclusion when the GAD-7 score is ≥ 10, even in the absence of clinically significant depressive symptoms.

For the Risk Group 2 (preterm birth) will be: (i) having given birth to a preterm infant, defined as gestational age < 37 weeks.

#### 2.1.3. Exclusion Criteria

The exclusion criteria will be: (i) severe cognitive impairment substantially limiting comprehension of study procedures or engagement in the intervention; (ii) acute psychiatric condition requiring immediate/intensive treatment (e.g., active psychosis or acute suicidality); (iii) neonatal medical instability or severe infant medical conditions that may interfere with participation or completion of assessments; (iv) current participation in other structured parenting interventions specifically focused on mentalization and/or video-feedback; (v) unable or unwilling to provide informed consent.

#### 2.1.4. Sample Size

Given the feasibility nature of the study, the planned sample size was selected to provide adequate information on recruitment, retention, intervention completion, assessment completion, acceptability, and study procedures. Although a minimum sample size of *N* = 28 was estimated, increasing to approximately *N* = 36 after allowing for 20% attrition, the planned sample size of 48 participants provides an additional margin to support the study’s feasibility objectives, e.g., [31,32].

#### 2.1.5. Recruitment

Potential participants for the VFI-RF study will be identified and recruited through participating Italian centers. Recruitment will be conducted through the following sources:

(i) Mothers with moderate-to-high anxiety and/or depressive symptoms will be recruited through a network of local and regional counselling and socio-health services (e.g., the Department of Mental Health, ASL Roma 1, Lazio Region).
(ii) Mothers of preterm infants will be recruited through the Neonatal Units and the Neonatal Intensive Care Units (NICUs) of Hospitals in the Abruzzo region
(iii) Public advertisements, including online announcements, local media, and flyers/pamphlets distributed in relevant community and healthcare settings.

A member of the research team will contact the heads of service (e.g., principals/directors), case managers, and/or other relevant staff within the participating organizations to provide information about the study. If the organization agrees to collaborate, staff will be asked to share written study information with individuals whom they consider potentially eligible. Interested individuals will then be contacted by a member of the research team. Alternatively, those who prefer to be approached directly by the research team may complete a “consent to contact” form authorizing a member of the team to get in touch. At this stage, it will be explicitly clarified that individuals are providing consent only to be contacted by the research team to discuss the study, rather than consenting to participate in the study itself. To maximize reach and accessibility, the study will also be promoted via online channels, local media, and posters/leaflets distributed through collaborating organizations. Interested individuals will then be able to contact the research team voluntarily to obtain further information.

Recruitment has not yet begun; we plan to begin participant recruitment in April 2026 and to complete it by the end of 2027.

#### 2.1.6. Procedure

Mothers who meet the inclusion criteria and voluntarily consent to participate will be sent a link to complete the baseline survey. Eligible mothers will complete the baseline assessment (T0) when their infants are approximately 3 months of age. Baseline measures will include self-report questionnaires assessing sociodemographic information, maternal reflective functioning, psychological distress, parenting stress, perceived social support, and attachment style. Mothers will then be invited to attend a virtual appointment (within the same week) to review and discuss baseline findings. Prior to the online meeting, a clinician with psychodynamic training will review the mother’s responses. During the first session, the clinician will conduct a clinical interview to clarify any ambiguous findings and to establish an initial therapeutic engagement. The intervention procedures will be agreed in detail with each participant. Specifically, mothers will be instructed on how to use the platform, a schedule of sessions will be established, and clear guidance will be provided on how to record the video, where and how to upload/send it, and the sequence of everyday caregiving routines to be filmed. During the video-feedback sessions, selected moments of mother–infant interaction (e.g., feeding, diaper changing) previously recorded by mothers and submitted to the researcher will be reviewed and discussed jointly.

The privacy and confidentiality of all video recordings will be strictly protected. Mothers will receive an individualized secure link to a dedicated cloud-based storage system accessible only to the researcher. Uploaded videos will be stored using a pseudonymized alphanumeric participant code. The code will be generated using the first two letters of the participant’s first name, the first two letters of the participant’s surname, and the last two digits of the participant’s year of birth. No full names or other directly identifying information will be included in the video file names.

Following completion of T0, mothers will participate in a structured mentalization-based video-feedback program delivered synchronously online over a five-month period, comprising ten fortnightly sessions. During the intervention, mothers will be asked to record brief videos (approximately 5 min) of everyday caregiving interactions with their infant following standardized instructions. Recordings will be shared securely with the clinician and reviewed during online sessions, in which selected interaction sequences will be collaboratively explored to promote maternal reflective functioning and sensitivity (see Section 2.1.7).

**Figure 1:**
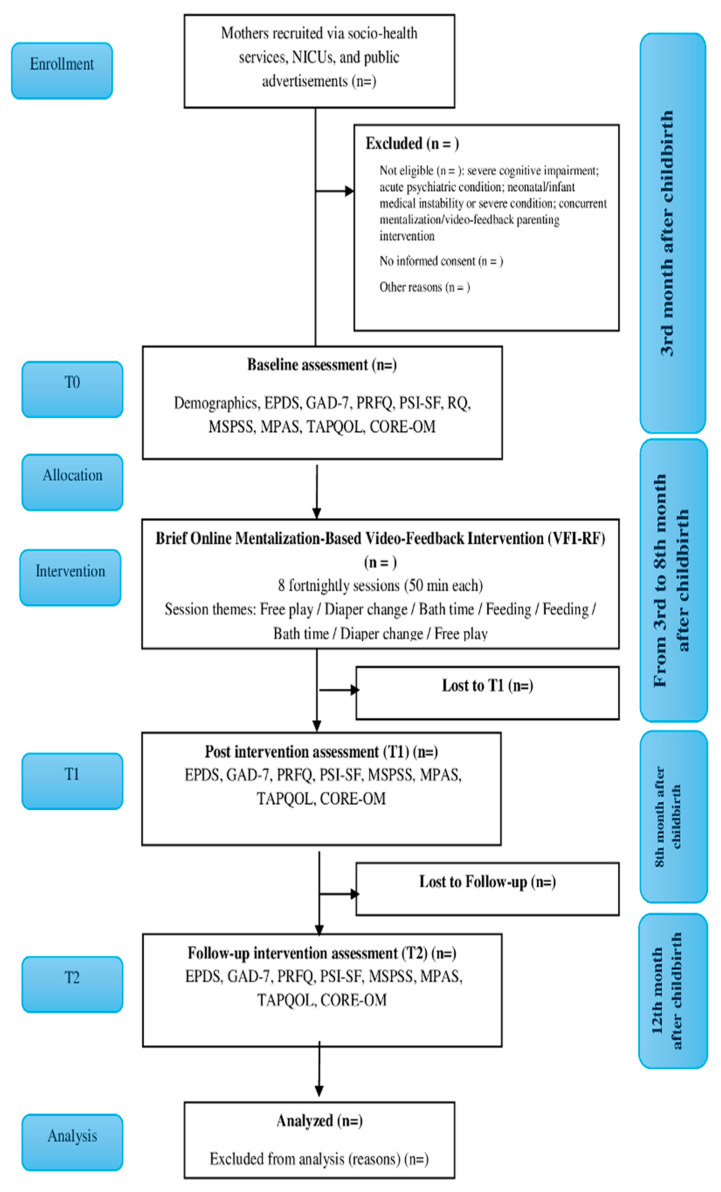
Participant timeline.

Post-intervention assessment (T1) will be completed when infants are approximately 8 months of age, using the same procedures as at baseline. A follow-up assessment (T2) will be conducted when infants are approximately 12 months old to examine the maintenance of effects over time. At both time points, participant feedback will also be collected to explore mothers’ experiences of and satisfaction with the VFI-RF (see Figure 1).

All assessments and intervention sessions will be conducted remotely via secure digital platforms (e.g., Qualtrics, Zoom, Teams), in accordance with data protection and ethical requirements. Clinicians and research staff involved in data collection and observational coding will follow standardized protocols to ensure consistency and reliability across time points and study sites.

#### 2.1.7. Description

##### Brief Online Mentalization-Based Video-Feedback Intervention (VFI-RF)

The intervention is a structured, brief, online VF program grounded in a psychodynamic and mentalization-based framework, specifically designed to enhance maternal reflective functioning within the mother–infant relationship. It comprises 8 + 2 sessions delivered synchronously over a 5-month period, spanning from when infants are approximately 3 months old to approximately 8 months of age.

The intervention will be delivered by a trained clinician within a psychodynamic framework. From this perspective, VFI conceptualizes the therapist as a mediator of meaning: through attuned reflection, emotional containment, and collaborative exploration, the clinician supports mothers in transforming implicit affective reactions into explicit, reflective understanding [33]. The therapeutic relationship therefore functions as a regulatory and interpretative scaffold, enabling mothers to develop more coherent representations of mother–infant interaction and to internalize more reflective ways of relating to their infant [34].

Phase 1. Initial Assessment and Therapeutic Engagement (Session 1). Session 1 will focus on clinical and relational assessment and on establishing the therapeutic frame. The study and intervention procedures will be introduced, the VF approach will be explained, and an initial therapeutic alliance will be fostered. This phase aims to create a secure, collaborative, and non-judgmental context, providing the foundation for subsequent reflective work. Clinicians and mothers also discuss the results of baseline assessment.

Phase 2. Video-Feedback Intervention Sessions (Sessions 2–9). The core of the intervention consists of VF sessions focused on everyday caregiving interactions. Mothers will be instructed to record brief videos (approximately 5 min) of routine interactions with their infant (i.e., free play, feeding, diaper changing, bathing) following standardized guidelines. Each session will typically include: (i) viewing the video sequence in its entirety; (ii) selecting and focusing on salient interactional moments, with attention to infant communicative cues and maternal responses; and (iii) clinician-guided reflective exploration, during which the clinician adopts a mentalizing stance and facilitates meaning-making. Across this phase, the clinician supports the mother in attending to and reflecting on her own emotional and mental states, recognizing the infant’s signals, needs, and intentions, and exploring moments of dyadic regulation as well as misattunement and repair. The emphasis is placed on fostering curiosity and reflective understanding rather than providing behavioral instruction. The intervention further conceptualizes the therapeutic relationship as an active mechanism of change, with the mother–clinician relational exchange supporting the development of mentalization and parental reflective functioning.

Phase 3. Final Restitution and Reflective Integration (Session 10). The final session will focus on reviewing the overall intervention process and reflecting on perceived changes in the mother–infant relationship. It will consolidate the reflective capacities developed across sessions and support the mother’s transition toward a more confident and reflective parenting stance. This phase aims to facilitate integration of the work completed during the program and to promote continuity of reflective caregiving beyond the intervention period.

Phase 4. Follow-up. Follow-up assessments will be conducted 12 months postpartum to examine the maintenance of intervention effects on clinical and psychological outcomes variables. In addition, qualitative data will be collected to explore mothers’ experiences of, and satisfaction with, the VFI-RF.

**Figure 2:**
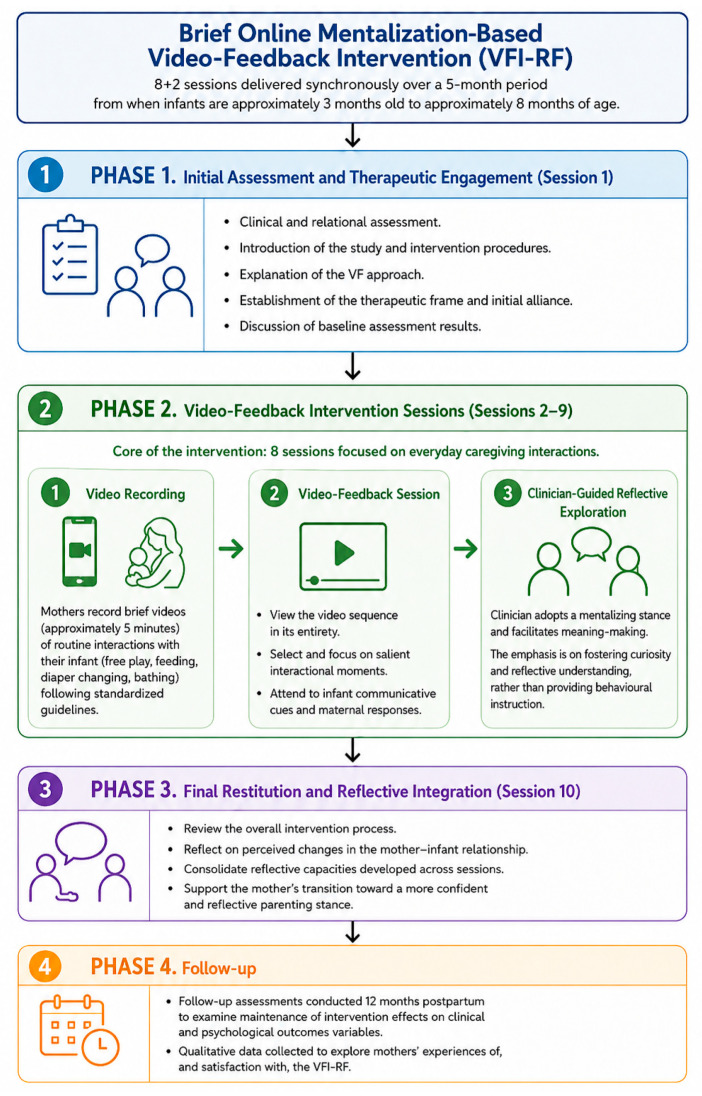
Structure and content of the VFI-RF.

A visual description of the VFI-RF phases is provided in Figure 2.

##### Dosage and Administration

The intervention will consist of eight VFI-RF sessions (approximately 50 min per session) delivered over 5 months. A two-week interval between sessions (Phase 2) was selected to allow mothers sufficient time to record brief everyday interactions, upload them securely to the clinician, and enable the clinician to review the material and discuss it within the research team prior to the session. All sessions will be delivered by the same trained psychologist, who works within a psychodynamic framework and has received specific training in the use of video-feedback. Participation is entirely voluntary, and participants may withdraw at any time and/or decline to take part in any specific intervention component. Pending the outcome of the study, the treatment manual will be available upon request.

### 2.2. Outcomes

#### 2.2.1. Feasibility and Acceptability

Feasibility and acceptability will be assessed using a mixed-methods approach, combining quantitative indicators with qualitative feedback from participants and service providers. Pre-specified progression criteria will be applied using a traffic-light system, as recommended in methodological guidance for pilot and feasibility trials [35,36]. Green criteria will indicate that progression to a definitive trial is feasible without major modifications, amber criteria will indicate that progression may be feasible with protocol amendments, and red criteria will indicate that progression would not be feasible without substantial redesign. Quantitative findings and qualitative feedback will be interpreted together, adopting a holistic multi-criteria approach, to inform decisions about the feasibility, acceptability, and optimization of the intervention and trial procedures (see Table 1).

#### 2.2.2. Clinical Outcome Measures—Self-Attribution

Parental Reflective Functioning Questionnaire (PRFQ) [4]; Italian version [37]. The PRFQ is an 18-item self-report measure of parental reflective functioning. It comprises three subscales: Interest and Curiosity (IC) (interest in the child’s mental states), Certainty about Mental States (CMS) (tendency toward overly certain/opaque interpretations of the child’s mind), and Pre-Mentalizing (PM) (non-mentalizing or maladaptive attributions about the child’s mental states). Items are rated on a 7-point Likert scale. The Italian validated version will be used and has shown acceptable-to-good internal consistency (PM α = 0.70; CMS α = 0.82; IC α = 0.74).

Edinburgh Postnatal Depression Scale (EPDS) [38] Italian version of [39]. The EPDS is a 10-item self-report screening tool for depressive symptoms in the perinatal period. The Italian validation reported good internal consistency (α = 0.79).

Generalized Anxiety Disorder Scale (GAD-7) [40]; Italian version [41]. The GAD-7 is a 7-item self-report measure assessing symptoms of generalized anxiety. Items are rated on an ordinal response scale, with higher scores indicating greater anxiety symptom severity. A cut-off score of ≥ 10 will be used to indicate probable moderate anxiety.

Parenting Stress Index–Short Form (PSI-SFI) [42] Italian version of [43]. The PSI-SF is a 36-item self-report questionnaire assessing parenting-related stress across three domains: Parental Distress (α = 0.80), Parent–Child Dysfunctional Interaction (α = 0.81), and Difficult Child (α = 0.72). Items are rated on a Likert scale, and a total parenting stress score can be derived (α = 0.89).

Multidimensional Scale of Perceived Social Support (MSPSS) [44]; Italian version [45]. The MSPSS is a 12-item self-report measure assessing perceived social support from Family, Friends, and a Significant Other. Items are rated on a 7-point Likert scale, with higher scores indicating greater perceived support. The Italian version demonstrates adequate reliability comparable to the original scale, with Cronbach’s α ranging from 0.87 to 0.91.

Relationship Questionnaire (RQ) [46]; Italian version [47]. The RQ is a single-item measure designed to evaluate adult attachment style. Respondents are presented with four short paragraphs describing prototypical attachment patterns—secure, fearful, preoccupied, and dismissing—and are asked to select the description that best represents their typical approach to close relationships.

Maternal Postnatal Attachment Scale (MPAS) ([48]; Italian version [49]). The MPAS is a 19-item self-report measure assessing the mother’s emotional bond with her infant in the postnatal period, including dimensions such as quality of attachment, absence of hostility, and pleasure in interaction. The Italian validation reported good internal consistency (α = 0.77).

TNO-AZL Preschool Children Quality of Life (TAPQOL) [50]. The TAPQOL is a 43-item parent-report questionnaire assessing health-related quality of life in young children (approximately 9 months to 6 years), covering multiple domains (e.g., sleep, mood, anxiety, problem behavior).

Clinical Outcomes in Routine Evaluation—Outcome Measure (CORE-OM) ([51]; Italian version [52]). The CORE-OM is a 34-item self-report measure of adult psychological distress and functioning, developed to monitor change over the course of psychological treatment. The Italian validation reported good internal consistency (α = 0.90) for the total score.

The timeline and the specific timing of each psychological assessment across the study waves are detailed in Table 2.

### 2.3. Data Analysis

Analyses will first focus on the primary feasibility and acceptability outcomes. Recruitment, eligibility, consent, intervention uptake, retention, assessment completion, missing data, and participant-reported acceptability will be summarized descriptively and evaluated against the pre-specified traffic-light progression criteria reported in Table 1. Indicators falling within the green range will suggest that progression to a future definitive trial is feasible without major modifications; amber indicators will suggest that progression may be feasible following protocol refinements; and red indicators will indicate that substantial modifications or redesign would be required before proceeding. Quantitative feasibility indicators will be interpreted together with qualitative feedback from participants and service providers to provide an integrated assessment of the feasibility and acceptability of the VFI-RF intervention and study procedures.

Exploratory analyses of secondary outcomes will examine changes from baseline (T0) to post-intervention (T1) and follow-up (T2). Linear mixed-effects models for repeated measures will be fitted separately for each secondary outcome, including assessment time as the main within-subject factor of interest. Maternal–infant risk profile will be included to account for heterogeneity in the sample and will be defined according to the presence of infant preterm birth, clinically significant anxiety and/or depressive symptoms, or the co-occurrence of psychosocial and infant-related risk factors.

The primary exploratory focus of these models will be on the effect of time, describing whether outcome scores change across assessment points, and on the time × risk profile interaction, exploring whether trajectories of change differ across risk subgroups. The main effect of risk profile will be considered only as an adjustment factor and descriptive indicator of baseline/sample heterogeneity, rather than as a primary inferential target.

Planned exploratory contrasts will estimate changes from T0 to T1 and from T0 to T2. Where appropriate, T1 to T2 contrasts will also be examined to describe whether observed changes appear to be maintained, reduced, or further increased over time. Effect estimates, confidence intervals, and descriptive patterns of observed change will be reported. Given the feasibility nature of the study, the expected small sample size, and the absence of a comparison group, all analyses of secondary outcomes will be interpreted cautiously as exploratory and hypothesis-generating only, and will not be considered definitive evidence of intervention effectiveness.

All statistical analyses will be conducted using SPSS Statistics version 30.0 (IBM Corp., Armonk, NY, USA).

### 2.4. Auditing

Written progress reports and project meetings will be convened at least quarterly, and more frequently as required. These updates will review enrolment, intervention development, recruitment rates, treatment fidelity, follow-up progress, data management procedures, and adherence to project timelines. Any issues identified will be discussed, and feasible mitigation strategies will be proposed and agreed upon. 

## 3. Discussion

The present study protocol aims to assess the feasibility and acceptability of an exclusively online mentalization-based video-feedback intervention designed to support maternal reflective functioning and the early mother–infant relationship and to identify the implementation requirements for its delivery in postnatal risk contexts. Consistent with its single-arm feasibility design, the study is not intended to evaluate causal effectiveness. Rather, it will examine whether the VFI-RF can be delivered, accessed, and received as intended in real-world postnatal risk contexts. The study will also generate implementation learning regarding referral pathways, recruitment, intervention uptake, retention, privacy procedures, participant burden, clinician delivery, and completeness of outcome assessment. It will also provide preliminary descriptive evidence on trajectories in maternal reflective functioning, psychological distress, parenting stress, perceived social support, maternal emotional bonding with the infant, and child health-related quality of life, with the purpose of assessing the suitability and sensitivity of these measures for a future randomized controlled trial rather than testing intervention effects.

The perinatal period is marked by substantial emotional, relational, and caregiving demands that may challenge mothers’ capacity to regulate their own emotions and to understand the infant’s behavior as meaningful and intentional. These challenges may increase the risk of disengaged or intrusive interaction patterns [33]. Maternal depressive symptoms, anxiety, parenting stress, younger maternal age, preterm birth, and broader relational difficulties have been associated with lower parental reflective functioning and less optimal mother–infant interaction quality [5,6,9]. Interventions aimed at strengthening reflective functioning during this sensitive developmental window are therefore highly relevant for prevention and early clinical support, particularly given evidence that parental reflective functioning plays a central role in sensitive caregiving, dyadic regulation, and children’s socio-emotional development [2,4,33]. In the present study, however, these theoretical premises serve as the rationale for the intervention model and outcome selection, not as hypotheses that can be causally tested within the current design.

Video-feedback interventions are typically brief, focused approaches in which mothers are actively supported to review everyday interactions with their infants. With the guidance of a clinician, these interactions become opportunities for reflection on the infant’s possible internal states and on the links between observable behavior and underlying emotions, intentions, and thoughts. In this way, VFIs may foster parental curiosity, enhance reflective functioning, and support more attuned caregiving [20,21]. A central objective of the present protocol is to determine whether these reflective processes can be acceptably supported in a fully online format. Exploration of feasibility and acceptability indices will help us in confirming it. Moreover, by examining preliminary trajectories in maternal reflective functioning and maternal emotional bonding with the infant following an online intervention, the study addresses the growing need for accessible and flexible support for mothers experiencing perinatal psychological vulnerability, while recognizing that such trajectories cannot be attributed to the intervention in the absence of a comparison group.

Previous studies have shown that video-feedback interventions can be adapted to a range of risk conditions, including socioeconomic disadvantage, maternal psychopathology, and relational difficulties [19,53,54]. In the present study, the inclusion of mothers with different postnatal risk profiles may help identify practical and clinical issues related to psychosocial vulnerability and infant-related medical risk. However, any comparison between these groups will be exploratory and interpreted with caution, given the nature of the study, the single-arm design, and the limited sample size. The study will therefore not determine for whom the intervention is most effective, but will provide preliminary information on whether recruitment, adherence, acceptability, and outcome assessment procedures are workable across different vulnerability profiles.

The longitudinal design further allows preliminary trajectories to be described from baseline to post-intervention and follow-up. These trajectories will be interpreted as descriptive and hypothesis-generating, rather than as evidence of intervention effects. Repeated assessments will also be used to evaluate the acceptability and completeness of outcome measurement over time and to inform the selection of clinically meaningful endpoints for a future controlled trial [18,24]. Therefore, follow-up data will be used to assess retention, data completeness, participant burden, and the suitability of repeated outcome assessment, rather than to draw conclusions about maintenance of treatment effects.

### Strengths and Limitations

Although the fully online delivery of the intervention may represent an important advantage for mothers whose personal, geographical, clinical, or caregiving circumstances limit access to in-person perinatal services, some limitations should also be acknowledged. The online format has the potential to increase accessibility and reduce logistical barriers while preserving the core reflective component of video-feedback work; however, it may also exclude mothers without suitable electronic devices, a stable internet connection, sufficient digital literacy, or access to a private space for participation.

Most importantly, the single-arm feasibility design does not allow causal conclusions regarding intervention effectiveness. Any observed trajectories over time may reflect infant maturation, spontaneous improvement in maternal distress, regression to the mean, repeated assessment, or nonspecific therapeutic factors, rather than the specific effects of the VFI-RF. The relatively small feasibility sample will also limit conclusions about differential responses across risk profiles or potential mechanisms of change. Accordingly, analyses of clinical outcomes, risk profiles, and reflective functioning will be interpreted as exploratory and hypothesis-generating.

In addition, reliance on self-report measures for some outcomes may introduce reporting biases, particularly among mothers experiencing psychological distress [7]. Self-selection bias may also occur, as mothers experiencing greater difficulties, or conversely those who are more motivated and available to engage in reflective work, may be more likely to participate. Finally, the home environment may vary considerably across participants, potentially affecting the quality, comparability, and emotional context of recorded interactions. Privacy may also be more difficult to ensure during online sessions, particularly in crowded households or when a quiet and confidential space is unavailable. These issues will be specifically monitored through feasibility indicators and qualitative feedback and will inform refinements to privacy procedures, clinician support, and strategies to reduce participant burden in a future randomized controlled trial.

## 4. Conclusions

This study protocol outlines a theoretically grounded and methodologically rigorous approach to evaluating the feasibility and acceptability of an online mentalization-based video-feedback intervention for mothers in the perinatal period. By focusing on enrolment, intervention uptake, retention, assessment procedures, and the acceptability of remote video-feedback delivery, the study addresses important practical and methodological questions regarding the implementation of VFI-RF in postnatal risk contexts. In addition to feasibility outcomes, the protocol will provide preliminary descriptive information on trajectories in maternal reflective functioning, psychological distress, parenting stress, perceived social support, maternal emotional bonding with the infant, and child health-related quality of life. The findings are expected to inform refinement of the VFI-RF protocol, optimization of study procedures, and identification of potential barriers and facilitators to implementation. More broadly, the study will provide evidence to support the design of a future randomized controlled trial evaluating the efficacy and clinical utility of online mentalization-based video-feedback interventions in perinatal populations.

## Figures and Tables

**Table 1:** Progression criteria for feasibility and acceptability.

Domain	Area	Indicator	Green	Amber	Red
Feasibility	Enrolment	Mothers referred	≥80% of target	60–79%	<60%
		Eligible among referred	≥60%	40–59%	<40%
		Consented among eligible	≥70%	50–69%	<50%
	Intervention uptake	VFI-RF sessions attended	≥75%	50–74%	<50%
	Retention	Baseline participation	≥90%	75–89%	<75%
		Follow-up participation	≥80%	65–79%	<65%
		Ineligibility/withdrawal reasons across study phases	Infrequent/ non-systematic	Modifiable reasons	Recurrent major issues
	Assessment/data quality	Assessments completed	≥85%	70–84%	<70%
		Missing data on key measures	≤10%	11–20%	>20%
Acceptability	Assessment procedures	Number/frequency acceptable	≥75% positive	50–74%	<50%
		Duration/content/mode acceptable	≥75% positive	50–74%	<50%
	Mothers’ experience	VFI-RF experience	Predominantly positive	Mixed, with modifiable concerns	Predominantly negative or recurrent major concerns
		Satisfaction with VFI-RF/study procedures	≥75% satisfied	50–74%	<50%

**Table 2:** Schedule of participant assessment.

	Pre-Intervention		Post-Intervention	
	Screening	Baseline (T0)	Post-Treatment (T1)	Follow-Up (T2)
Demographics (e.g., age, education, employment and marital status), clinical history, and psychosocial risk variables	✔	✔		
Maternal reflective functioning (PRFQ)		✔	✔	✔
Maternal depression (EPDS)	✔	✔	✔	✔
Maternal anxiety (GAD-7)	✔	✔	✔	✔
Maternal Parenting stress- Short Form (PSI-SF)		✔	✔	✔
Maternal Perceived social support (MSPSS)		✔	✔	✔
Relationship Questionnaire (RQ)		✔		
Maternal postnatal attachment (MPAS)		✔	✔	✔
Child’s emotional and behavioral development (TAPQOL)		✔	✔	✔
Clinical Outcomes in Routine Evaluation- Outcome Measure (CORE-OM)		✔	✔	✔
Satisfaction questionnaire ad hoc			✔	✔

## Data Availability

The data generated and/or analyzed during this study will be made available by the corresponding author upon reasonable request, after publication of the results and in accordance with applicable regulations on privacy, informed consent, and confidentiality. SPIRIT checklist was available as Supplementary Materials (Table S1).

## Supplementary Materials

The following supporting information can be downloaded at: https://www.mdpi.com/article/10.3390/jcm15135271/s1, Table S1: SPIRIT 2025 checklist of items to address in a randomized trial protocol.

## Abbreviations

The following abbreviations are used in this manuscript:

PRF	parental reflective functioning
VFI-RF	mentalization-based video-feedback intervention
VF	video-feedback
VFIs	video-feedback interventions
NICUs	Neonatal Intensive Care Units
PRFQ	Parental Reflective Functioning Questionnaire
IC	Interest and Curiosity
CMS	Certainty about Mental States
PM	Pre-Mentalizing
EPDS	Edinburgh Postnatal Depression Scale
GAD-7	Generalized Anxiety Disorder Scale
PSI-SF	Parenting Stress Index–Short Form
MSPSS	Multidimensional Scale of Perceived Social Support
RQ	Relationship Questionnaire
MPAS	Maternal Postnatal Attachment Scale
TAPQOL	TNO-AZL Preschool Children Quality of Life
CORE-OM	Clinical Outcomes in Routine Evaluation—Outcome Measure
GDPR	General Data Protection Regulation
